# Sequestosome1/p62 protects mouse embryonic fibroblasts against low-dose methylercury-induced cytotoxicity and is involved in clearance of ubiquitinated proteins

**DOI:** 10.1038/s41598-017-17112-8

**Published:** 2017-12-01

**Authors:** Yasukazu Takanezawa, Ryosuke Nakamura, Ryohei Harada, Yuka Sone, Shimpei Uraguchi, Masako Kiyono

**Affiliations:** Department of Public Health, School of Pharmacy, Kitasato University, 5-9-1 Shirokane, Minato-ku, Tokyo, 108-8641 Japan

## Abstract

Methylmercury (MeHg) is a widely distributed environmental pollutant that causes a series of cytotoxic effects. However, molecular mechanisms underlying MeHg toxicity are not fully understood. Here, we report that sequestosome1/p62 protects mouse embryonic fibroblasts (MEFs) against low-dose MeHg cytotoxicity via clearance of MeHg-induced ubiquitinated proteins. p62 mRNA and protein expression in MEFs were temporally induced by MeHg exposure p62-deficient MEFs exhibited higher sensitivity to MeHg exposure compared to their wild-type (WT) counterparts. An earlier and higher level of accumulation of ubiquitinated proteins was detected in p62-deficient cells compared with WT MEFs. Confocal microscopy revealed that p62 and ubiquitinated proteins co-localized in the perinuclear region of MEFs following MeHg treatment. Further analysis of MEFs revealed that ubiquitinated proteins co-localized with LC3-positive puncta upon co-treatment with MeHg and chloroquine, an autophagy inhibitor. In contrast, there was minimal co-localization in p62-deficient MEFs. The present study, for the first time, examined the expression and distribution of p62 and ubiquitinated proteins in cells exposed to low-dose MeHg. Our findings suggest that p62 is crucial for cytoprotection against MeHg-induced toxicity and is required for MeHg-induced ubiquitinated protein clearance.

## Introduction

Mercury released into aquatic environments by natural events and various anthropogenic activities is readily methylated by microorganisms^1^. Methylmercury (MeHg) causes serious damage to various organs in both animals and humans. Bioaccumulation of MeHg occurs within the food chain, and consumption of contaminated fish and other aquatic seafood is the primary source of exposure to humans^2^. Severe neurological disorders occur in victims of MeHg poisoning, which is the cause of Minamata disease^3^. Several studies have evaluated the effects of toxic or subtoxic doses of MeHg *in vivo* and *in vitro* ^4–7^. However, the molecular responses and effects of low doses of MeHg to which humans are exposed through daily food intake are not well-understood. Thus, our previous work focused on the effects of low-dose MeHg *in vivo* and *in vitro*.

We recently reported that exposure to low levels of MeHg in a Th2 allergic response model dose not affect the Th2 immune response in mice^8^. Moreover, we recently showed that a low dose of MeHg activates autophagy in several cell types, and the lack of autophagy gene 5 (*Atg5*) makes cells more sensitive to MeHg toxicity^9^. These findings suggest an association between autophagy and protective mechanisms against low-dose MeHg toxicity.

Macroautophagy (hereafter referred to as “autophagy”) is a major catabolic process that delivers cellular contents, including damaged proteins, protein aggregates, and organelles, to lysosomes for degradation and recycling^10^. Autophagy occurs constitutively at low levels, but is accelerated by a variety of cellular stresses, such as nutrient starvation, accumulation of abnormal proteins, and damaged organelles^11^. Autophagic degradation has the ability to be selective for its cargo via ubiquitin signaling, thereby removing only damaged cellular contents. Selective autophagy is coordinated by autophagy receptors linking cargos tagged with ubiquitin chains to the autophagosomal membrane. Recently, several studies have focused on autophagy in MeHg-exposed cells. The increased microtubule-associated protein 1 light chain (LC3)-II/LC3-I ratio and increased autophagic vacuoles in human neuronal stem cells after MeHg exposure suggest possible upregulation in autophagic activity^12^. Inducing autophagy with rapamycin led to cytoprotection, whereas suppression of autophagy with chloroquine exacerbated cytotoxicity after MeHg exposure. This suggested that autophagy was protective against MeHg-induced cytotoxicity^13^.

The sequestosome (SQSTM1)/p62 protein is a well-known autophagy receptor that binds LC3 and ubiquitinated proteins in the early process of autophagy, and localizes to sites of autophagosome formation^14^. It is believed that p62 plays a fundamental role in clearing protein aggregates, damaged mitochondria, peroxisomes, and invading microbes through selective autophagy^15–17^. In addition, p62 is an inducible protein in response to various stressors^18,19^.

There has been an exponential increase in interest in the key role of p62 in the pro-survival function of autophagy against various stresses. Our recent study showed that the p62 protein was increased in response to MeHg during activation of autophagy^9^. However, the underlying mechanism and role of this protein has not been explored in regard to MeHg toxicity. The present study aimed to elucidate the involvement of p62 in MeHg detoxification in mouse embryonic fibroblasts (MEFs). Here, we report that MeHg induces p62 expression and the accumulation of ubiquitinated proteins in MEFs. Our findings suggest a protective role of p62 against MeHg-induced toxicity and that p62 is involved in preventing the accumulation of MeHg-induced ubiquitinated proteins.

## Results

### MeHg induces SQSTM1/p62 protein levels and the formation of p62 aggregates

**Figure 1:**
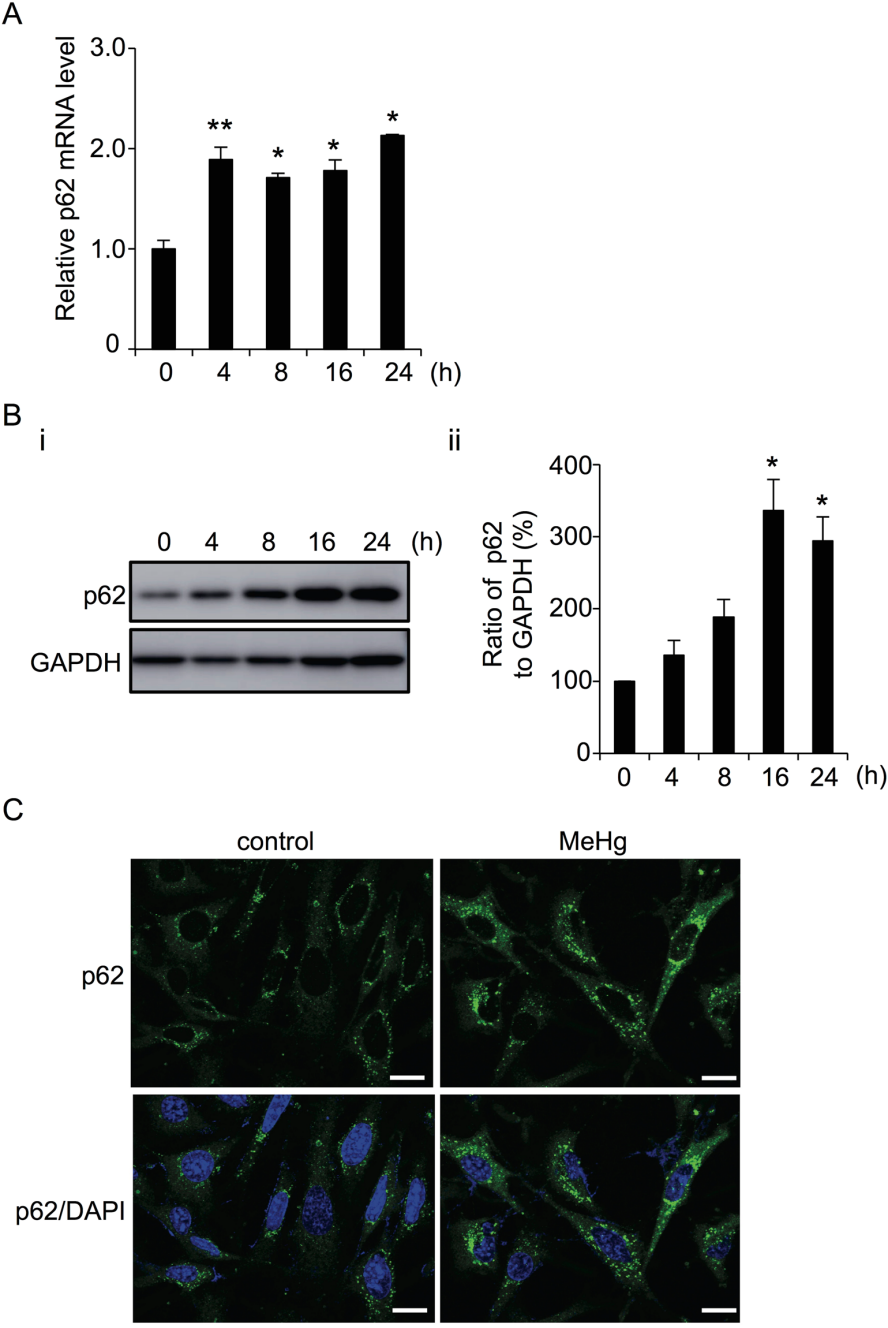
Methylmercury (MeHg) leads to increased p62 mRNA and protein levels in mouse embryonic fibroblasts (MEFs). (**A**) mRNA levels of p62 relative to GAPDH after treatment with 1 µM MeHg for 0-24 h. Data are expressed as means ± SEM for 3 independent experiments. **p < 0.01. (**B**) Protein levels of p62 were determined by immunoblotting of cells treated with 1 µM MeHg for 0-24 h. GAPDH was used as the loading control (i). Blots were quantitated and relative expression values determined (ii). Data are expressed as means ± SEM for 3 independent experiments. *p < 0.05; **p < 0.01. (**C**) Wild-type (WT) MEFs were treated with or without 1 µM MeHg for 24 h, and then fixed with paraformaldehyde. Endogenous p62 was immunostained with an anti-p62 antibody. Cells were analyzed with a confocal microscope. Scale bars: 20 µm.

SQSTM1/p62 interacts with LC3 and is a substrate for autophagy. Stimulation of autophagy typically decreases cellular p62 levels^20^. In our previous study, however, we showed that MeHg stimulated autophagy in MEF cells, but increased the p62 protein levels in several types of cells such as MEFs, Caco-2, and SH SY-5Y^9^. Hence, we first assessed the effect of MeHg on p62 mRNA expression. Induction of p62 mRNA was observed at 4 h after 1 µM MeHg treatment and the levels were maintained for 24 h (Fig. 1A). p62 protein levels increased in a time-dependent manner with the peak occurring at 16 h (Fig. 1B). Consistent with this finding, confocal microscopic analysis of MeHg-treated MEFs also showed high p62 protein levels at 24 h (Fig. 1C).

**Figure 2:**
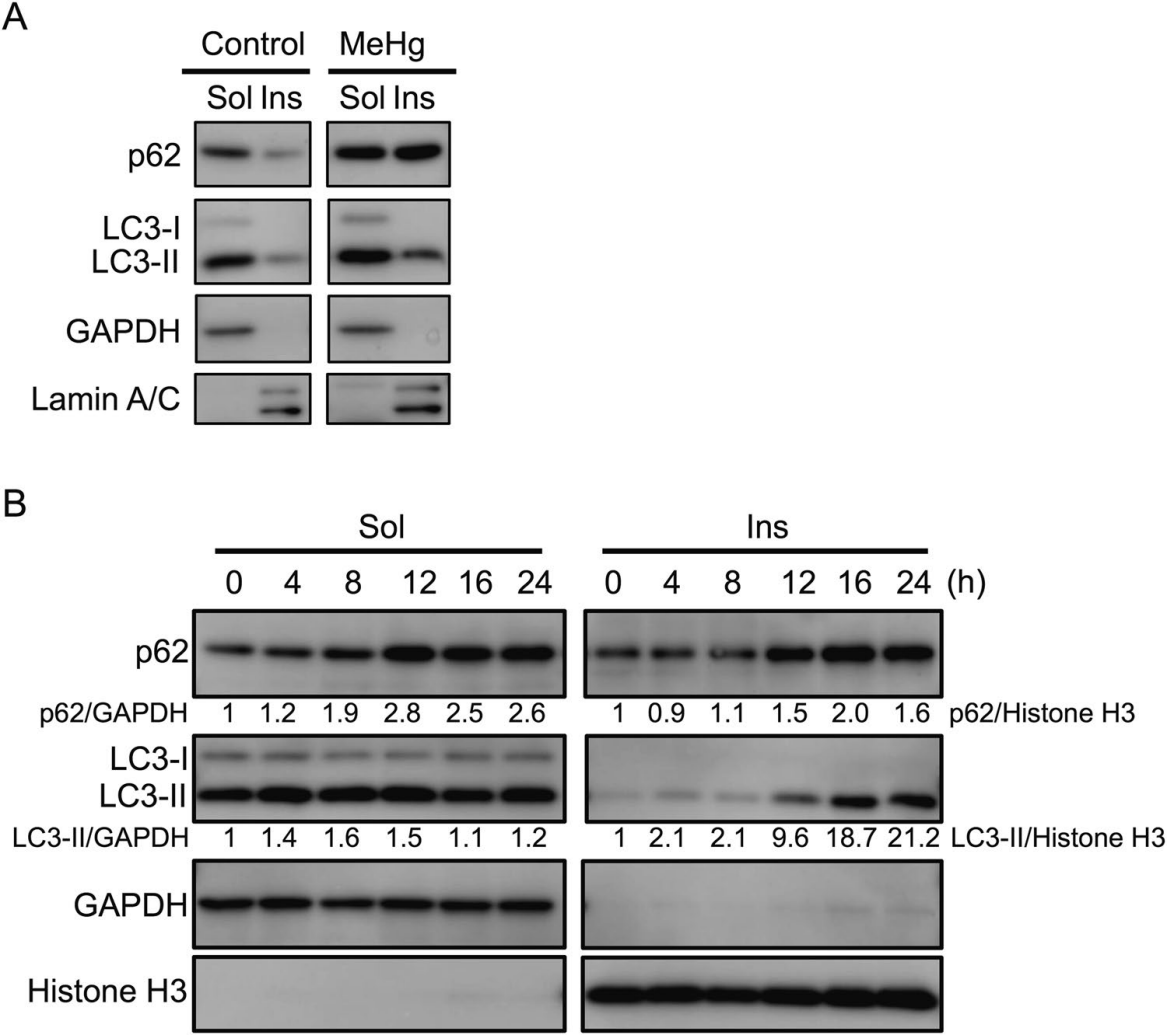
Methylmercury (MeHg) increases p62 protein levels in both the cytosol and aggregate fractions of mouse embryonic fibroblasts (MEFs). p62, LC3-I, and LC3-II protein levels in the soluble (Sol) and insoluble (Ins) fractions from wild-type (WT) MEFs. (**A**) Cells were treated with or without 1 µM MeHg for 24 h. GAPDH and lamin A/C were used as loading controls. (**B**) Cells were treated with 1 µM MeHg for 0–24 h. GAPDH and histone H3 were used as loading controls.

To better understand the effects of autophagy on p62 expression, we then investigated whether p62 induction after 1 µM MeHg exposure correlated with the accumulation of p62 aggregates. This study involved separating the soluble cytosolic proteins from the insoluble aggregate fraction (Fig. 2A). MeHg-treated cells displayed a clear increase in p62 in both the cytosolic and insoluble aggregate fractions. Concurrent with the increase in p62, MeHg also increased LC3-II protein levels in both fractions.

Subsequently, we examined whether the expression pattern of p62 was associated with that of LC3-II (Fig. 2B). Expression of p62 increased following MeHg treatment in both the cytosolic (peak 12 h) and aggregate (peak 16 h) fractions. Importantly, the expression pattern of the LC3-II protein differed from that of p62 in the aggregate fraction.

**Figure 3:**
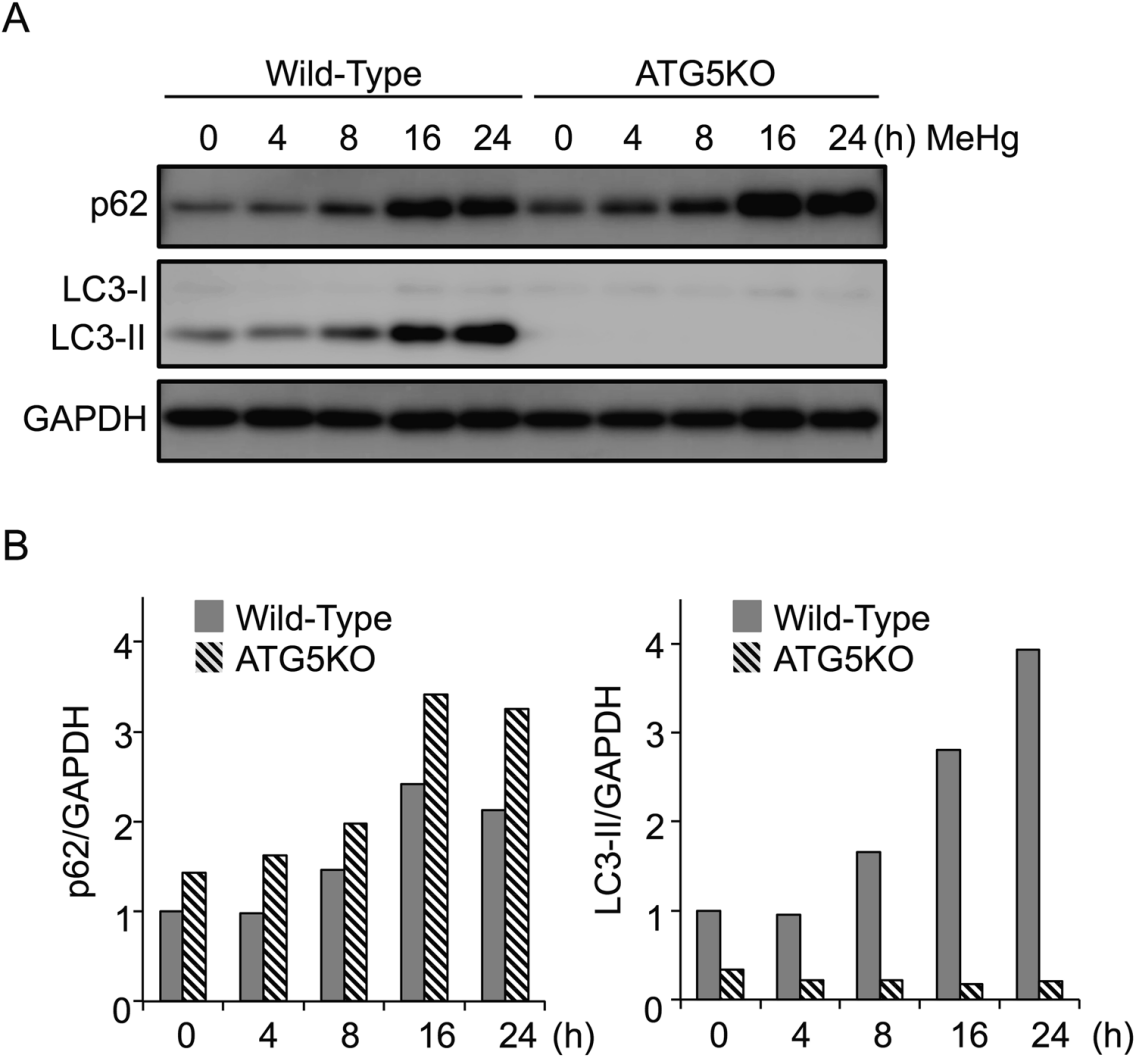
Methylmercury (MeHg) increases p62 protein in an autophagy-independent manner. (**A**) Wild-type (WT) and Atg5 knockout (KO) mouse embryonic fibroblast cells were treated with 1 µM MeHg for 0–24 h. Whole cell lysates were subjected to immunoblots for p62, LC3-I, and LC3-II. GAPDH was used as the loading control. (**B**) Blots were quantitated and relative expression values determined.

Further, we tested p62 expression in autophagy-deficient cells, Atg5KO MEFs. Treatment with MeHg increased p62 levels temporally, peaking at 16 h in wild-type MEFs. Similarly, in Atg5KO MEFs, p62 protein levels increased upon MeHg exposure. However, the accumulation was much greater in Atg5KO MEFs than in wild-type MEFs (Fig. 3). LC3-I levels were low and unchanged, and LC3-II levels were very low in Atg5KO MEFs, with or without MeHg treatment (Fig. 3). These findings indicate that upregulation of p62 by MeHg is independent of autophagic activity. However, as LC3-II levels increased, p62 levels decreased subsequently in WT cells, compared to Atg5 KO MEFs, suggesting that MeHg activates autophagy and degrades p62 at later time points.

### Clearance of ubiquitinated proteins is impaired in the absence of p62

**Figure 4:**
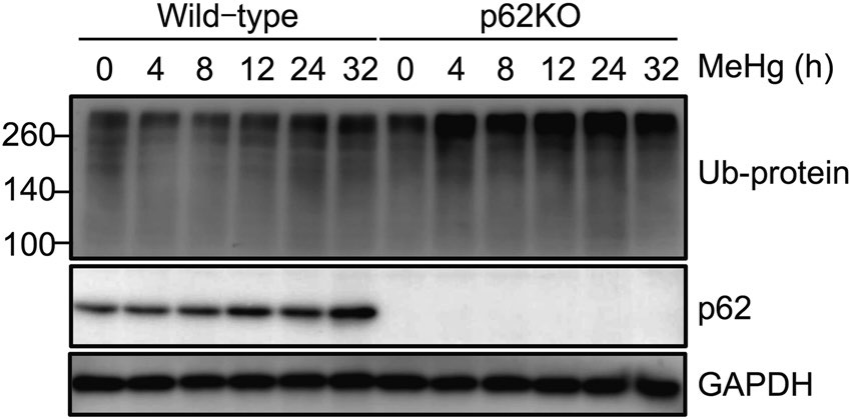
p62 deficiency increases the levels of ubiquitinated proteins after methylmercury (MeHg) treatment. Wild-type and p62-defecient mouse embryonic fibroblasts were seeded in 6-cm dishes for treatments. After treatment with 1 µM MeHg for 0–24 h, whole cell lysates were immunoblotted with anti-ubiquitin and p62 antibodies. GAPDH was used as the loading control.

p62 binds ubiquitin non-covalently both *in vitro* and *in vivo* ^21,22^. To assess the function of p62 under MeHg exposure, we examined whether MeHg facilitated protein ubiquitination in MEFs via immunoblot analysis. Treatment of wild-type MEFs with 1 µM MeHg increased the quantity of ubiquitinated proteins temporally (Fig. 4). The extent of this accumulation in p62 knockout (p62KO) MEFs occurred earlier than that observed in wild-type MEFs.

### Co-localization of p62 and ubiquitinated proteins

**Figure 5:**
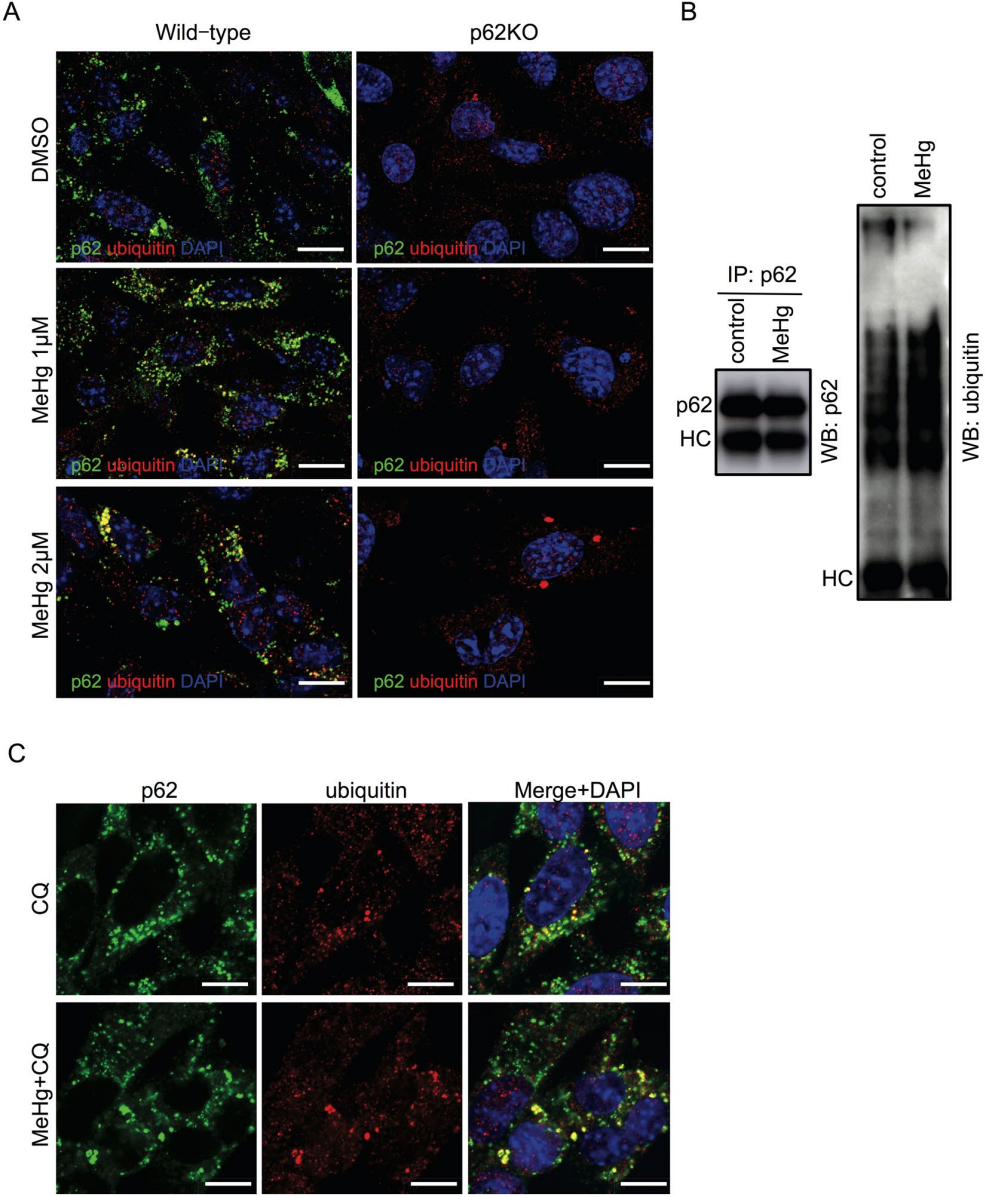
Methylmercury (MeHg) induces ubiquitinated proteins and co-localizes with p62. (**A**) Confocal immunofluorescence images of wild-type and p62 knockout (KO) mouse embryonic fibroblasts (MEFs) stained for p62 (green) and ubiquitinated (red) proteins. Scale bars: 20 µm. (**B**) Wild-type MEFs were lysed with Triton X-100 buffer and 1 mg of protein was immunoprecipitated with a p62 antibody. The interaction was determined by immunoblot analyses for p62 and ubiquitin. (**C**) Confocal immunofluorescence images of wild-type MEFs stained for p62 and ubiquitin. Cells were treated with 20 µM chloroquine (CQ) for 6 h or with 1 µM MeHg for 18 h and 20 µM CQ for 6 h. Scale bars: 20 µm.

Because p62 contains a ubiquitin-associated domain that recognizes substrates targeted for degradation, we next investigated whether ubiquitinated proteins were co-localized with p62 after MeHg treatment. Immunofluorescence staining revealed that p62 was present in numerous round bodies in the perinuclear area while ubiquitinated proteins were located in the cytoplasm, with weak staining, in wild-type MEFs (Fig. 5A). In wild-type MEFs treated with 1 µM MeHg, we observed an increased number of p62-positive puncta in the perinuclear area, and a substantial proportion of p62 appeared to co-localize with the ubiquitinated proteins. Similar immunofluorescence staining of ubiquitinated proteins was seen in p62KO MEFs. Notably, robust accumulation of ubiquitinated proteins was seen upon 2 µM MeHg treatment (Fig. 5A).

To confirm the interaction between p62 and ubiquitinated proteins, cell lysates from MEFs with or without MeHg treatment were immunoprecipitated using an antibody against p62. MeHg treatment increased the interaction of p62 with ubiquitinated proteins (Fig. 5B). To further investigate the relationship between p62 and ubiquitinated proteins, cells were treated with chloroquine (CQ), a lysosomotropic agent inhibiting the autophagic flux, leading to an accumulation of ubiquitinated proteins. In cells treated with CQ, there was an extensive accumulation of p62 and ubiquitinated proteins in the perinuclear area. Following co-treatment of CQ with MeHg, ubiquitinated proteins were strongly co-localized with p62 (Fig. 5C).

### The loss of p62 impairs co-localization of ubiquitinated proteins with LC3 puncta

**Figure 6:**
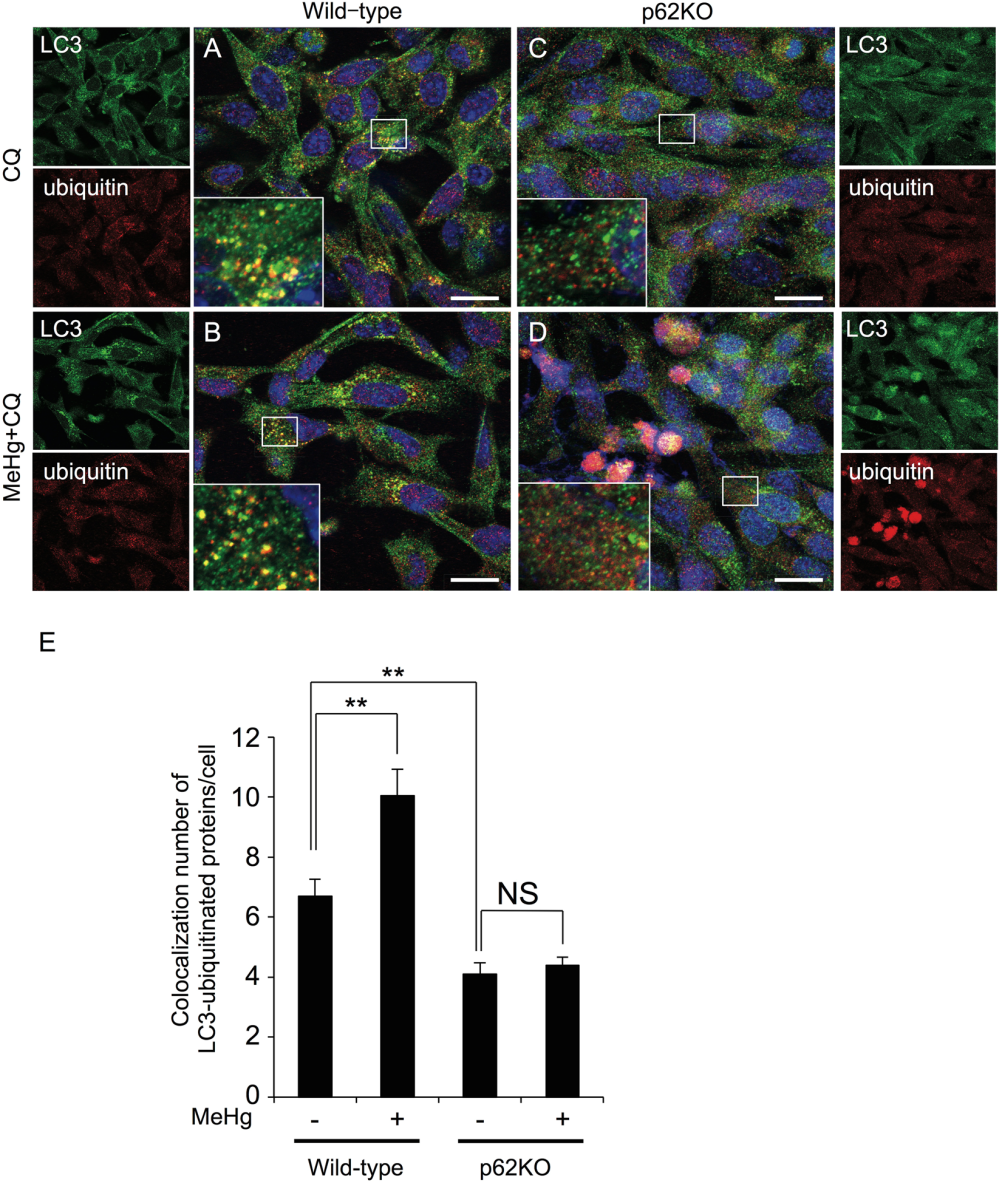
Co-localization of LC3 with ubiquitinated proteins is impaired in the absence of p62. Wild-type and p62 knockout (KO) mouse embryonic fibroblasts (MEFs) were immunostained for endogenous LC3 (green) and ubiquitinated proteins (red), and imaged using confocal microscopy. WT and p62KO MEFs were treated with 20 µM chloroquine (CQ) for 6 h (**A**,**C**) or with 1 µM MeHg for 18 h before incubation with 20 µM CQ for 6 h (**B**,**D**). The boxed areas in the composite images are shown at a higher magnification. Scale bars: 20 µm. (**E**) Quantification of the number of colocalizations was performed in 20 cells. Results are shown as mean ± SEM.

p62 is believed to mediate the clearance of ubiquitinated proteins by autophagy via direct interaction with LC3 on the membrane through the LC3-interacting region. To confirm the involvement of p62 in MeHg-induced autophagy, we examined the distribution of ubiquitinated proteins and LC3 puncta in wild-type and p62KO MEFs. To clarify the distribution of LC3 and ubiquitinated proteins, cells were treated with the autophagy inhibitor, CQ. CQ elevated levels of ubiquitinated proteins that were mainly located in the perinuclear region of the cell, and mostly co-localized with LC3 in wild-type MEFs (Fig. 6A). The co-localization of ubiquitinated proteins and LC3 was enhanced in wild-type MEFs treated with 1 µM MeHg and CQ (Fig. 6B). In contrast, the number of ubiquitinated proteins overlapping with LC3 was substantially decreased in p62KO cells (Fig. 6C). Upon MeHg treatment, despite the abundant accumulation of ubiquitinated proteins, p62KO MEFs showed limited co-localization of these proteins with LC3 (Fig. 6D).

### Involvement of p62 in protection against MeHg-induced toxicity

**Figure 7:**
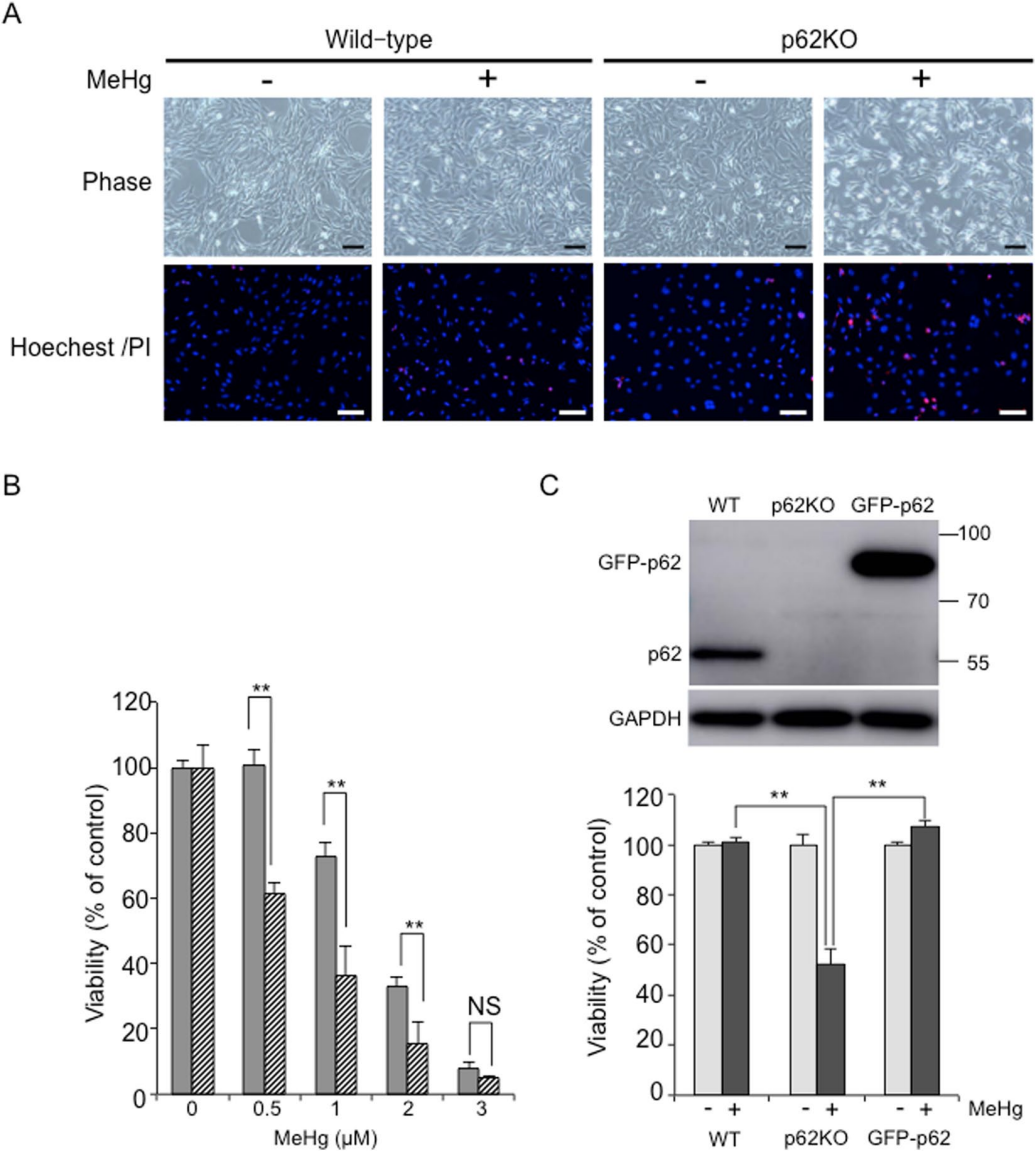
p62 knockout (KO) mouse embryonic fibroblasts (MEFs) are more sensitive to methylmercury (MeHg) than wild-type MEFs. (**A**) Wild-type and p62KO MEFs were treated with 1 µM MeHg for 24 h then stained with Hoechest 33342 and propidium iodide. Images were captured using confocal microscopy. Hoechest 33342 labeled the cell nuclei blue and propidium iodide labeled dead cells red. Scale bars: 100 µm. (**B**) WT and p62KO MEFs were exposed to various concentrations of MeHg for 24 h. Viability was determined using a CCK-8 assay kit. Closed and hatched bars represent for wild-type and p62KO MEFs, respectively. Values are expressed as means ± SEM (n = 3). *p < 0.05; **p < 0.01. (**C**) WT, p62KO and GFP-p62KO MEFs were treated with 0.5 µM MeHg for 10 h. The total amount of p62 in the lysates was determined in the immunoblots (Upper panel). Cell viability was determined using a CCK-8 assay kit (Lower panel). Values are expressed as mean ± SEM (n = 4). *p < 0.05; **p < 0.01.

To determine whether p62 had a protective role, we assessed the sensitivity of wild-type and p62KO MEFs to MeHg. Numerous rounded and/or shrunken cells were observed in MeHg-exposed p62KO MEFs. These are typical features of apoptotic cell death. Wild-type MEFs showed fewer cells with such morphological features (Fig. 7A). In addition, the cytotoxicity of MeHg in wild-type and p62KO MEFs was examined using the CCK-8 viability assay (Fig. 7B). Consistent with the Hoechest 33342/PI data, at each concentration (0.5–3 µM), p62KO MEFs had a lower survival rate than wild-type MEFs following 24 h of MeHg treatment. Furthermore, the GFP-p62 was transfected into p62KO MEFs using a retroviral expression system, and cell viability was assessed after MeHg exposure. As expected, GFP-p62 most probably fully compensated for p62 (Fig. 7C). These results suggest that p62 protects cells from MeHg-induced cell death.

## Discussion

Autophagy is a conserved cellular process that generally protects cells by eliminating damaged, nonfunctional, or aggregated proteins and organelles. Autophagy plays a critical role in maintaining cellular homeostasis and protects cells from various environmental stressors. Among these, the role of autophagy against heavy metal stress has been little understood despite their adverse effects on cell function and viability. Recently, we reported that low-dose MeHg exposure promoted autophagy, and Atg5-dependent autophagy served to protect cells from MeHg cytotoxicity^9^. SQSTM1/p62 is an autophagic receptor and delivers ubiquitinated cargoe for autophagic degradation^11,23^, implying that p62 is involved in MeHg-induced autophagy. In the current study, we showed that p62KO MEFs exhibited an enhanced sensitivity to low-dose MeHg toxicity, demonstrating our hypothesis that p62 is another key molecule in MeHg-induced cytotoxicity.

To understand how p62 contributes to cell survival against MeHg toxicity, we first examined response of p62 to MeHg in wild-type MEFs. Levels of p62 mRNA (Fig. 1A) and protein (Fig. 1B–D) were upregulated temporally after exposure to 1 µM MeHg. These results indicated that p62 was not only a substrate of autophagy, but also an inducible protein, and that upregulation occurred through a transcriptional event in response to low-dose MeHg exposure. We observed in Atg5KO MEFs that p62 protein levels increased after MeHg exposure (Fig. 3), indicating that increased p62 levels upon MeHg exposure were not derived from an inhibition of autophagic activity. Moreover, we showed in wild-type MEFs treated with MeHg that similar levels of p62 protein were observed in both the 1% Triton X-100 soluble and insoluble fractions (Fig. 2A,B). We found that extensive accumulation of LC3-II occurred in the insoluble fraction upon treatment with MeHg. These results indicate that MeHg-induced p62 forms protein aggregates immediately with LC3-II.

p62 directly binds to ubiquitinated proteins and plays a role in the formation and clearance of protein aggregates. The ubiquitin-proteasome system (UPS) and the autophagy-lysosome pathway are the main degradative pathways for intracellular ubiquitinated proteins. p62 is involved in both processes, wherein it mediates the delivery of ubiquitinated proteins^24,25^. This present results show that MeHg induced ubiquitinated proteins temporally and suggests that p62 is involved in their clearance. The accumulation of ubiquitinated proteins occurred earlier and to a greater extent (Fig. 4) and robust accumulation of ubiquitinated proteins was observed in p62KO MEFs rather than in WT cells (Fig. 5A), suggesting that p62 is involved in the clearance of ubiquitinated proteins induced by MeHg exposure. Furthermore, ubiquitinated proteins were co-localized with LC3 in wild-type MEFs (Fig. 6A and B), while the extent of overlap between ubiquitinated proteins and LC3 was decreased in p62KO MEFs (Fig. 6C and D). Therefore, p62 is probably involved in the degradation of MeHg-induced ubiquitinated proteins, via autophagy. To assess the contribution of UPS, we investigated the effect of a proteasome inhibitor, MG132. Co-treatment with MeHg and MG-132 resulted in increased accumulation of ubiquitinated proteins in insoluble fractions, rather than treatment with only MG132 (Fig. S1). Moreover, we detected insoluble ubiquitinated proteins of approximately 260 kD prominently in p62KO MEFs, suggesting that p62 is involved in the clearance of MeHg-induced ubiquitinated proteins via the UPS. Together, our findings suggest that not only autophagy but also the UPS contributes to clearance of MeHg-induced ubiquitinated proteins, and p62 plays important roles in their clearance via autophagic and the proteasomeal pathways.

Herein, p62KO MEFs exhibited an enhanced sensitivity to MeHg exposure compared to their wild-type counterparts (Fig. 7A and B) and exogenous GFP-p62 was able to compensate for their loss of p62 (Fig. 7C), thereby strongly suggesting the significance of p62 in protecting cells from MeHg toxicity. However, mechanisms underlying the enhanced sensitivity of p62 MEFs to MeHg remain unclear. Since p62KO MEFs showed excess accumulation of ubiquitinated proteins by MeHg treatment, at least in part, they may have undergone MeHg-induced cell death. Furthermore, other possibilities of enhanced MeHg sensitivity of p62KO MEFs cannot be excluded, as p62 has multiple functions. p62 was shown to interact with Kelch-ECH-associated protein 1 (KEAP1) at the transcription factor NF-E2-related factor 2 (NRF2) binding site, thereby promoting NRF2 release from KEAP1^26–28^. NRF2 activation induced the expression of many antioxidant genes^29^. p62 also interacts with tumor necrosis factor receptor-associated factor (TRAF6) and activates NF-κB^30^. It has been known that NF-κB promotes tumor cell survival through its ability to reduce reactive oxygen species (ROS) during transformation in cancer tissues^31^. In addition to accumulation of ubiquitinated proteins, as reported previously, MeHg induces oxidative stress through an increase in intracellular ROS, such as peroxide and superoxide anions^32^. Further studies are needed to investigate the molecular mechanisms underlying the role of p62 in regulating the NRF2 and TRAF6 pathways and in ROS production by MeHg exposure.

The present results show that increased levels of p62 interact with ubiquitinated proteins and play an essential role in their turnover following MeHg exposure. Neuronal inclusions contain aggregates of p62 and ubiquitinated proteins in patients with various neurodegenerative disorders such as Parkinson’s disease and amyotrophic lateral sclerosis^33^. Similar neuronal changes have been observed in various animal models of MeHg exposure^34,35^, and MeHg was suggested to be a risk factor for other neuronal disorders such as Alzheimer’s disease^36^ and ALS^37^. However, the relation between p62 and ubiquitinated proteins induced by MeHg, and these neurodegenerative diseases, has not been established. To further define the effect of MeHg on neurodegenerative diseases, it is necessary to investigate the role of p62 during low-dose and long periods of MeHg exposure, especially through daily food intake, and understand the coordination between the development of neurodegenerative diseases and the p62-dependent degradation of ubiquitinated proteins.

In conclusion, this study reported, for the first time, the protective role of the selective autophagy receptor p62 against low-dose MeHg exposure, and its role in the clearance of MeHg-induced ubiquitinated proteins. In future, we aim to further characterize the mechanisms underlying p62-mediated ubiquitinated proteins are degraded to protect against MeHg toxicity. Understanding the roles and mechanisms underlying the regulation of low-dose MeHg-induced ubiquitinated proteins could offer interesting novel therapeutic approaches for neurodegenerative diseases.

## Materials and Methods

### Chemicals

MeHg chloride (115-09-3) was purchased from Tokyo Kasei (Tokyo, Japan). Dimethyl sulfoxide (041-29351) was purchased from Wako Pure Chemical (Osaka, Japan). Chloroquine diphosphate (C6628) was purchased from Sigma-Aldrich (St. Louis, MO, USA).

### Cell Culture

Immortalized *Atg5* ^−/−^ and *Atg5* ^+/+^ MEFs (kind gifts from N. Mizushima) were maintained in Dulbecco’s modified Eagle’s medium (Sigma-Aldrich, D5796) supplemented with 10% heat-inactivated fetal bovine serum (Tissue Culture Biologicals, Seal Beach, CA, USA), 100 units/ml penicillin, 100 µg/ml streptomycin, and 292 µg/ml L-glutamine (Thermo Fisher Scientific, Rockford, IL, USA, 10368-016). Immortalized *p62* ^−/−^ and *p62* ^+/+^ MEFs (gifted by M. Komatsu and T. Yanagawa) were cultured in Dulbecco’s modified Eagle’s medium (Sigma-Aldrich, D6546) supplemented with 10% fetal calf serum, 0.1 mM non-essential amino acids (Thermo Fisher Scientific, 11140-050), 1 mM sodium pyruvate (Thermo Fisher Scientific, 11360-070), 100 units/ml penicillin, 100 µg/ml streptomycin, and 292 µg/ml L-glutamine. All cell lines were maintained at 37 °C in a 5% CO_2_ humidified atmosphere. All experiments were performed in subconfluent, exponentially growing cells that never exceeded passage number 25.

### Generation of GFP-p62 transfected cells

Retroviruses were generated using GP2-293 cells and a VSVenv-G protein encoding plasmid (pVSVenv-G) (Clontech, Palo Alto, CA, USA). Cells at 30% confluency (60-mm dish) were transfected with 1.8 µg of pMXs-puro GFP-p62 (addgene, Cambridge, MA, UK) and 1.8 µg of pVSVenv-G using 2 µl of lipofectamine 2000 (Life Technologies). At 24 h after transfection, the transduced cells were cultured in complete growth medium. At 96 h after transfection, culture medium was centrifuged for 10 min at 500 × g and stored at −80 °C. To generate GFP-p62 transfectants, p62KO MEFs were combined with polybrene (8 µg/ml) and 700 µl of culture medium containing retroviruses. At 24 h, transduced cells were cultured in complete growth medium with 3 µg/ml puromycin. After 2 days, puromycin-resistant cells were used for the MTT assay.

### Immunoblotting analysis

Cells were seeded in 6-cm dishes 24 h prior to treatment. After treatment, cells were washed in ice-cold phosphate-buffered saline (PBS), then lysed by radioimmunoprecipitation assay buffer (20 mM Tris pH 7.4, 0.1% sodium dodecyl sulfate (SDS), 1% sodium deoxycholate, 1% Nonidet P40, and protease/phosphatase inhibitor cocktail (Cell Signaling Technology, Danvers, MA, USA, 5872 S)), and sonicated for 10 sec on ice. For separation experiments, cells were lysed by Triton X-100 buffer (1% Triton X-100, 20 mM Tris pH 7.4, 137 mM NaCl, 2 mM EDTA, protease/phosphatase inhibitor cocktail) and centrifuged at 15,000 × g for 10 min. The supernatant and pellet were collected, and the protein concentrations measured using a bicinchoninic assay kit (Thermo Fisher Scientific, 23227) and an iMark microplate reader (Bio-Rad, Hercules, CA, USA).

For western blotting, cells were lysed in Laemmli sample buffer (SDS sample buffer with 286 mM 2-mercaptoethanol) and boiled at 95 °C for 5 min. Proteins (10 µg) were separated by SDS polyacrylamide gel electrophoresis (PAGE) and analyzed through western blot analysis. Briefly, samples were separated using 7.5–15% Tris-glycine gels and transferred onto polyvinylidene difluoride membranes (EMD Millipore, Billerica, MA, IPVH00010) using a Trans-Blot transfer apparatus (Bio-Rad). To avoid non-specific binding, membranes were blocked with Tris-buffered saline that contained 0.1% (w/w) Tween 20 (TTBS) and 5% (w/v) skim milk at 25 °C for 1 h. Further, the membranes were incubated with primary antibodies diluted in blocking solution at 4 °C overnight. The following primary antibodies were used: anti-p62 (Enzo Life Sciences, Farmingdale, NY, USA, PM045, 1:1000), anti-LC3B (Sigma-Aldrich, L7543, 1:1000), anti-ubiquitin (Cell Signaling Technology, #2118, 1:1000), anti-glyceraldehyde 3-phosphate dehydrogenase (GAPDH) (Cell Signaling Technology, #2118, 1:2000), anti-histone H3 (Cell Signaling Technology, #9715, 1:1000), and anti-lamin A/C (Merck Millipore, Temecula, CA, USA, #05-714, 1:1000). Membranes were subsequently incubated for 1 h with appropriate horseradish peroxidase-conjugated secondary antibodies (anti-mouse: GE Healthcare, Buckinghamshire, England, UK, NA931V, 1:6000; anti-rabbit: GE Healthcare, NA934V, 1:6000) in 5% skim milk in TTBS. Clarity enhanced chemiluminescence reagent (Nakalai Tesque, San Diego, CA, USA, 02230-30) was used to visualize proteins on an Amersham Imager 600 (GE Healthcare). Quantification of blots was carried out using ImageQuant TL 8.1 (GE Healthcare).

### Immunoprecipitation analysis

After treatment, cells were lysed by Triton X-100 buffer and centrifuged at 15,000 × g for 10 min. The protein concentration in the supernatant was measured using the bicinchoninic assay kit. Equal amounts of protein (1 mg) were incubated with 15 µl of the p62 antibody at 4 °C for 4 h, and then with 40 µl of 50% protein A Sepharose beads (GE Healthcare, 17078001) overnight at 4 °C. The beads were washed three times with Triton X-100 buffer. The protein complexes were eluted by boiling the beads in SDS-PAGE sample buffer and subjected to SDS-PAGE.

### Confocal microscopy

Cells were grown in 6-well plates on sterile cover slips. Following treatment, cells were fixed with 4% paraformaldehyde in PBS at 25 °C for 10 min. They were then transferred to a membrane permeabilization solution (0.2% Triton X-100) for 10 min. Cells were blocked in PBS that contained 1% bovine serum albumin for 1 h. For p62, LC3B, and ubiquitin staining, cells were incubated with anti-p62 (1:400), anti-LC3B (1:200), and anti-ubiquitin (1:400) antibodies at 25 °C for 1.5 h. Subsequently, cells were reacted with appropriate secondary antibodies labeled with Alexa Fluor 488 or 568 (Abcam, 1:1000) for 30 min. After three washes in PBS, cells were mounted using SlowFade Diamond (Thermo Fisher Scientific, S36964). For Hoechst 33342/propidium iodide (PI) staining, cells were seeded in 6-cm dishes 24 h prior to treatment. Following treatment, cells were incubated with 5 µg/ml Hoechst 33342 (Dojindo, H342) and 10 µg/ml PI (Thermo Fisher Scientific, P3566) for 10 min. Cells were then washed three times in PBS and mounted. All images were captured using a confocal laser scanning microscope (Zeiss LSM 710, Göttingen, Germany). Images were processed using LSM software ZEN 2012 (Zeiss).

### Cell viability assay

Cell viability was assessed by the WST assay using Cell Counting Kit-8 (Dojindo, Kumamoto, Japan) in accordance with the manufacturer’s instructions. Briefly, MEFs were seeded onto 96-well plates (1 × 10^4^ cells/well). After treating with different concentrations of MeHg (0, 1, 2, 3, and 4 µM) for 24 h, 10 µl CCK-8 solution was added to the wells and incubated for an additional 2 h at 37 °C. The number of viable cells was assessed by measuring the absorbance at 450 nm using an iMark microplate reader.

### RNA purification and the real-time quantitative polymerase chain reaction (RT-qPCR)

Total RNA was extracted from MEFs using a NucleoSpin RNA kit (Macherey-Nagel, Bethlehem, PA, 740955) and reverse-transcribed using the High-Capacity cDNA Reverse Transcription kit (Thermo Fisher Scientific, 4368814). The RT-qPCR was performed using FastStart SYBR Green Master (Roche, Indianapolis, IN, 04673484001) in a CFX-96 thermal cycler system (Bio-Rad) in accordance with manufacturer’s protocol. Triplicate samples were assessed for each gene of interest, and GAPDH was used as the control gene. Relative expression levels were determined by the 2^−∆∆Ct^ method. The primers used are given in Table S1.

### Statistics

Data were mean ± SEM. Statistical significance (p < 0.05) for each variable was estimated by analysis of variance (ANOVA) followed by a Tukey’s post-hoc analysis.

## Electronic supplementary material

Supplementary information
