# Supplementary material for: Sequestosome1/p62 protects mouse embryonic fibroblasts against low-dose methylercury-induced cytotoxicity and is involved in clearance of ubiquitinated proteins
Source: Sci Rep. 2017 Dec 1;7:16735. doi: 10.1038/s41598-017-17112-8 (PMC5711938; doi:10.1038/s41598-017-17112-8)
Supplement: Supplementary file 1 — Supplementary information [file 41598_2017_17112_MOESM1_ESM.pdf]

Supplementary information

Sequestosome1/p62 protects mouse embryonic fibroblasts against low-dose methylercury-induced cytotoxicity and is involved in clearance of ubiquitinated proteins

Yasukazu Takanezawa, Ryosuke Nakamura, Ryohei Harada, Yuka Sone, Shimpei Uraguchi, Masako Kiyono*

*To whom correspondence should be addressed.

Tel: +81-3-5791-6264

Fax: +81-3-3442-4146

E-mail: kiyonom@pharm.kitasato-u.ac.jp

Supplementary Table S1

Table S1. Primer sequences used for quantitative RT-PCR

mRNA targets	Oligonucleotides (5'→3')	Product Size (bp)
p62	F: GTGGGACAGCCAGAGGAACA<br>R: GCCCTTCCGATTCTGGCAT	136
GAPDH	F: AAATGGTGAAGGTCGGTGTG<br>R: TGAAGGGGTCGTTGATGG	108

Supplementary Figure S1

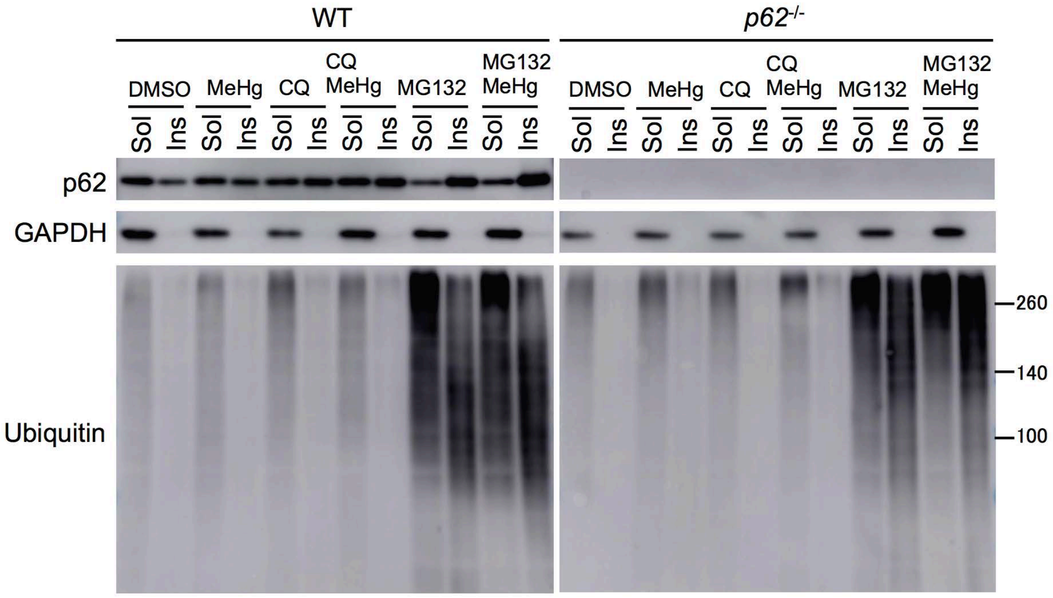

Supplementary Fig. S1. Autophagy or proteasome inhibitors enhanced accumulation of ubiquitinated proteins.

WT and p62KO MEFs were treated with 1 µM MeHg for 18 h and then 20 µM CQ or 10 µM MG132 was added for 6 h. Cells were lysed by Triton X-100 buffer and detergent-soluble and -insoluble fractions were harvested after centrifugation at 15,000 ×g for 10 min. Samples were immunoblotted with anti-p62 and ubiquitin antibodies. GAPDH was used as the loading control for the soluble fractions.
